# Comparison of the effects of the GPIIb-IIIa antagonist Zalunfiban and the P2Y12 antagonist Selatogrel on Platelet Aggregation

**DOI:** 10.1007/s11239-023-02867-x

**Published:** 2023-08-10

**Authors:** Benjamin J. Curry, A.O.F. Sem Rikken, C. Michael Gibson, Christopher B. Granger, Arnoud W.J. van ‘t Hof, Jurriën M. ten Berg, Lisa K. Jennings

**Affiliations:** 1 MLM Medical Labs, 140 Collins Street, Memphis, TN 38117 USA; 2 St. Antonius Hospital, Nieuwegein, The Netherlands; 3 Cardiovascular Research Institute Maastricht (CARIM), Maastricht, The Netherlands; 4 Boston Clinical Research Institute, Boston, MA USA; 5 Department of Cardiology, Duke University School of Medicine, Durham, NC USA; 6 MUMC+, Maastricht, The Netherlands; 7 Department of Cardiology, Zuyderland Medical Centre, Heerlen, The Netherlands; 8 University of Tennessee Health Science Center, Memphis, TN USA

**Keywords:** Zalunfiban, Selatogrel, Platelet, Glycoprotein IIb-IIIa, P2Y12

## Abstract

Understanding the pharmacodynamic effects of platelet inhibitors is standard for developing more effective antithrombotic therapies. An example is the antithrombotic treatment of acute coronary syndrome (ACS), in particular ST-elevated myocardial infarction (STEMI) patients who are in need for rapid acting strong antithrombotic therapy despite the use of aspirin and oral P2Y12-inhibitors. In this study, we evaluated two injectable platelet inhibitors under clinical development (the P2Y12 antagonist selatogrel and the GPIIb-IIIa antagonist zalunfiban) that may be amenable to pre-hospital treatment of STEMI patients. Platelet reactivity was assessed at inhibitor concentrations that represent clinically relevant levels of platelet inhibition (IC20-50%, 1/2Cmax, and Cmax). Light transmission aggregometry (LTA), was used to evaluate the initial rate of aggregation (primary slope, PS) and maximal aggregation (MA). Both adenosine diphosphate (ADP) and thrombin receptor agonist peptide (TRAP) were used as agonists. Zalunfiban demonstrated similar inhibition of platelet aggregation when blood was collected in PPACK or TSC, whereas selatogrel demonstrated greater inhibition in PPACK. In this study, using PPACK anticoagulant, selatogrel and zalunfiban affected PS in response to ADP equivalently at all drug concentrations tested. In contrast, zalunfiban had significantly greater potency at its Cmax concentration compared to selatogrel using TRAP as agonist. Upon evaluation of MA responses at lower doses, selatogrel had greater inhibition of MA in response to ADP than zalunfiban; however, at concentrations that represent Cmax, the drugs were equivalent. Zalunfiban also had greater inhibition of MA in response to TRAP at the Cmax dose. These data suggest that zalunfiban may provide greater protection in reducing thrombus formation than selatogrel, especially since thrombin is an early, key primary agonist in the pathophysiology of thrombotic events.

## Key Points

- Zalunfiban and selatogrel are effective inhibitors of ADP-induced platelet aggregation.
- Zalunfiban, compared to selatogrel, is a more potent inhibitor of TRAP-induced platelet aggregation.
- Zalunfiban and selatogrel pharmacodynamics should be evaluated in PPACK anticoagulant when using ADP as the agonist.
- Additionally, zalunfiban pharmacodynamics can also be evaluated in 3.2% TSC for MA only when using TRAP as the agonist.
- Zalunfiban may provide greater protection in reducing thrombus formation, since thrombin is an early agonist in the pathophysiology of thrombotic events.

## Introduction

Anti-platelets agents are the routine treatment in patients with acute coronary syndrome (ACS) to reduce recurrent thrombotic events. Various agents have targeted thromboxane A2, P2Y12 or protease-activated receptor 1 (PAR-1) as key activation pathways that regulate glycoprotein IIb-IIIa (GPIIb-IIIa) activation, fibrinogen binding and the subsequent platelet crosslinking that culminates in thrombus formation [1–7]. While current standard of care, dual anti-platelet therapy (aspirin and a P2Y12 antagonist), has proven to be effective in reducing the incidence of recurrent ischemic events in ACS [3, 4, 7], the use of an oral P2Y12 inhibitor has a delayed onset of antiplatelet effects and prehospital treatment has not led to improved patency [8–12]. Thus, there is a need for rapidly acting strong inhibitors which can be easily given in the ambulance to improve patency, reduce thrombus burden and to mitigate further atherothrombotic events [13–15].

Although P2Y12 inhibition by prasugrel or ticagrelor – and less effective clopidogrel – have been demonstrated to reduce adverse ischemic outcomes following ACS [3, 4, 7, 12, 16], residual atherothrombotic risk remains. This risk may be attributed to activation of the thrombin receptors, PAR-1 and PAR-4, that are mostly preserved despite robust P2Y12 inhibition. In line with this theory, a recent paper reported that even with adequate P2Y12 inhibition by the more potent agents, ticagrelor or prasugrel, ACS patients had normal platelet aggregation via PAR-1 and PAR-4 activation pathway [15].

While parenteral GPIIb-IIIa inhibitors, that provide broad and potent platelet inhibition, demonstrated robust clinical outcomes in the treatment of ACS [17–21], they require intravenous administration. Furthermore, their duration of effect may have led to increased bleeding and thrombocytopenia [22]. Thus, an easily administered, short-acting GPIIb-IIIa antagonist, such as zalunfiban, that can be injected subcutaneously in the ambulance while transporting the patient to the cardiac catheterization lab undergoing primary percutaneous coronary intervention (PCI) is preferable to reduce thrombotic events with an acceptable bleeding risk [23, 24]. A similar rationale using selatogrel, an injectable P2Y12 antagonist that inhibits platelet aggregation to ADP and has a faster onset of action compared to oral P2Y12 inhibitors, was also investigated in patients with chronic coronary syndromes [25]. However, these agents may differ in their ability to reduce thrombus formation in patients with STEMI. To date, data have been presented to demonstrate that sub-cutaneous administration of zalunfiban effectively inhibits platelet aggregation both to ADP and to TRAP, but data on the capability of selatogrel to inhibit TRAP-induced aggregation have not been provided while ADP induced aggregation is reduced [24, 25].

The purpose of this study is to compare the inhibitory properties of zalunfiban and selatogrel regarding inhibition of platelet aggregation and to assess which anticoagulant agents should be used for pharmacodynamic (PD) testing. This in vitro spiking study using human blood samples provides a greater understanding of how targeted drugs may affect the rate and extent of thrombus burden as we evaluate the potential clinical efficacy of these agents, especially for STEMI or ACS patients en route to hospital.

## Methods

### Study subjects and blood collection

Approximately 120 mL of whole blood (WB) was collected from consenting, healthy donors (n = 10) 18–65 years old (WIRB protocol #1,126,673). All donors denied taking aspirin or other medications known to affect platelet function within 2 weeks prior to blood collection. WB from each donor was collected in two separate anticoagulants in a 9:1 ratio of blood to anticoagulant (v/v) during the same venipuncture: 3.2% sodium citrate (TSC) anticoagulant (0.32% final concentration) or D-Phe-Pro-Arg- chloromethyl ketone dihydrochloride (PPACK) anticoagulant (100 µM final concentration). Previous studies reported that selatogrel activity is affected by TSC anticoagulant divalent cation chelation and reduces its potency [26, 27]. In contrast, zalunfiban was previously reported to have increased potency in 3.8% TSC [28, 29]. In this study, selatogrel and zalunfiban inhibitory effects were evaluated in parallel in PRP prepared from each anticoagulant to remove preanalytical variables that could impact drug performance, allowing direct comparisons in both conditions.

### Preparation of test compounds

Zalunfiban and selatogrel were provided by CeleCor Therapeutics. Stock solutions of each drug were prepared fresh from powder on each testing day. The working concentrations of zalunfiban (0, 25, 75, and 150 µg/mL) and selatogrel (0, 50, 250, and 500 µg/mL) were diluted from stock solutions prior to individual treatments with donor PRP. Zalunfiban was evaluated at 0, 25, 75 and 150 ng/mL, and selatogrel was evaluated at 0, 50, 250, and 500 ng/mL. Vehicle controls for zalunfiban (saline) and selatogrel (DMSO) were diluted equally in PRP for a final concentration of 0.1% (v/v). These treatments reflect the concentrations that achieve three clinically relevant scenarios: ~20–50% inhibitory concentration (IC20-50%), 1/2 Cmax, and Cmax according to published clinical data [24, 25].

### Preparation of PRP and treatment with test compounds

Platelet-rich plasma (PRP) was prepared from TSC or PPACK anticoagulated WB by centrifugation at 150 x G for 15 min at room temperature (RT). After PRP retrieval, platelet-poor plasma (PPP) was prepared by centrifuging the residual blood at 2,500 x g for 20 min at room temperature (RT). The platelet count in PRP was adjusted to 250,000/µL using the autologous PPP. The adjusted PRP was treated in separate tubes with each test compound at the indicated final concentrations, placebo, or nothing for 15 min at RT. Untreated PRP samples prepared from each anticoagulant were tested at the beginning and end of each set of donor experiments to confirm no loss of platelet function over the testing period.

WB collected into TSC anticoagulant was used for a series of preliminary experiments to determine if there were differences in platelet inhibition when zalunfiban was added to WB vs. PRP. The same experiments were not performed with selatogrel. For these studies, approximately half of the WB volume collected was treated directly with zalunfiban (0, 25, 75, and 150 ng/mL final concentration), mixed by gentle inversion, and incubated for 15 min at RT. The remaining half of unaltered WB was maintained at RT. The treated WB and the remaining untreated WB were centrifuged to prepare adjusted PRP, as described above. The adjusted PRP from the untreated WB received identical zalunfiban treatments for 15 min at RT. The samples were used to evaluate platelet reactivity using light transmission aggregometry (LTA) as described below.

### Platelet aggregation assays

Platelet aggregation tests were completed within 4 h of blood collection using Bio/Data PAP-8E instruments with a stirring speed of 1200 RPM [30]. An untreated and adjusted PRP sample was used to screen for aspirin ingestion by performing an aggregation test with arachidonic acid (Bio/Data Corporation; 1.6 mM final concentration). A maximal aggregation (MA) value ≥ 40% was considered a passing test demonstrating an aspirin effect is not present, and the donor sample was used for the study.

After treatment with the test compounds, the adjusted PRP for each donor was immediately added to aggregation cuvettes with a stir bar and transferred to the incubation wells (37 °C) of the aggregometer without stirring. The samples were allowed to warm to 37 °C for 3 min prior to moving the cuvette to the testing wells. Cuvettes were transferred to the testing wells, and the baseline was established for ~ 30 s. ADP or TRAP (200 µM) were diluted 1:10 in the adjusted, drug treated PRP, for a final concentration of 20 µM. The aggregation responses were recorded for at least 10 min after agonist addition. The TSC and PPACK sample aggregations were performed on two different instruments and the order of the treatments was inverted across the 8 channels to eliminate possible individual channel differences across the instruments. All MA results generated by the PAP-8E instrument above 100% were reported as 100%. Results were reported as the rate of aggregation or primary slope (PS; LTU/min) and the extent of aggregation or maximal aggregation (MA; %) for n = 10 donors as the mean ± standard deviation (SD). Additionally, results were analyzed as percent (%) inhibition for each drug treatment and donor using the formula: % Inhibition = (Vehicle – Drug Treatment)/Vehicle*100. These results were also reported as the mean ± SD for n = 10 donors. A two-way analysis of variance (ANOVA) with Tukey’s method for multiple comparison was used to compare ADP or TRAP mediated aggregation responses for zalunfiban or selatogrel across the range of drug concentrations evaluated. The same statistics were also used to compare the results in the presence of either TSC or PPACK to determine the impact of anticoagulant choice on the drug performance. Šidák correction was used to compare ADP or TRAP mediated aggregation responses for zalunfiban and selatogrel directly at IC20-50%, 1/2 Cmax and Cmax (GraphPad Prism Version 9.5.0).

## Results

Control aggregation experiments were performed with untreated PRP before and after aggregation experiments with drug-treated PRP to ensure platelet function was consistent throughout the testing of each subject’s samples. There were no differences in platelet aggregation response before and after each series of platelet aggregation tests; consequently, any differences in platelet function after either zalunfiban or selatogrel treatments were the result of the drug treatments and not due to altered platelet response over the duration of the testing period (data not shown).

**Fig. 1:**
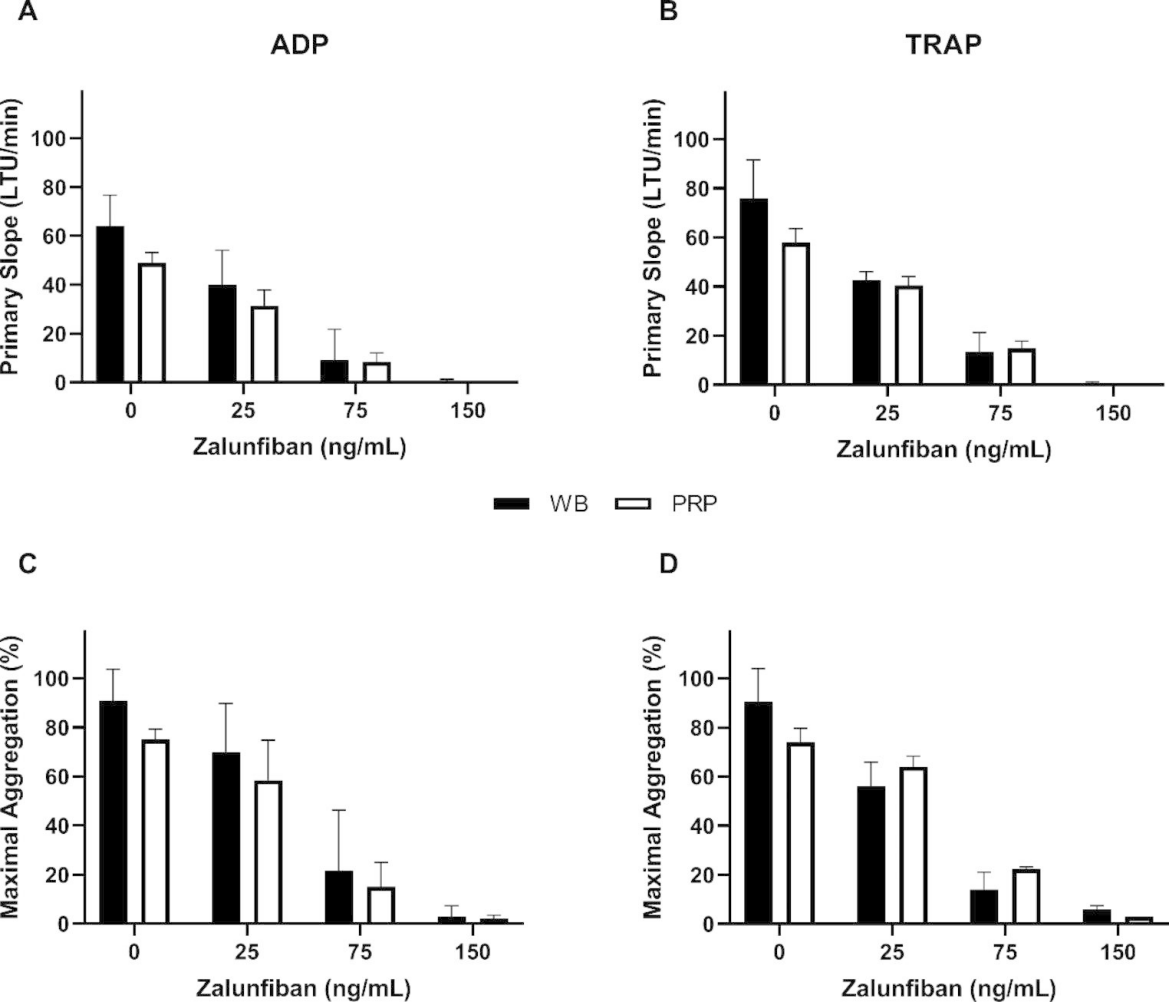
**Platelet aggregation with whole blood and PRP treatment with zalunfiban**. Whole blood collected from healthy donors (n = 2) was treated directly (black bars) with zalunfiban or used to isolate PRP, then treated with zalunfiban (white bars). Platelet aggregation was initiated with ADP (A and C) or TRAP (B and D) agonists. Results are presented as the primary slope (A and B) and maximal aggregation (C and D) mean ± SD.

Initially, anticoagulated WB treated directly with either vehicle controls or increasing concentrations of zalunfiban was compared to treatment with isolated PRP. This evaluation was performed as most studies with zalunfiban utilized whole blood testing with the VerifyNow platform [24], whereas PRP aggregation data were available using LTA for selatogrel [25]. Therefore, these same experiments were not performed with selatogrel. The PS and MA were measured in both treatment preparations, and zalunfiban-mediated aggregation inhibition was similar regardless if the drug was added to WB or PRP (Fig. 1).

**Fig. 2:**
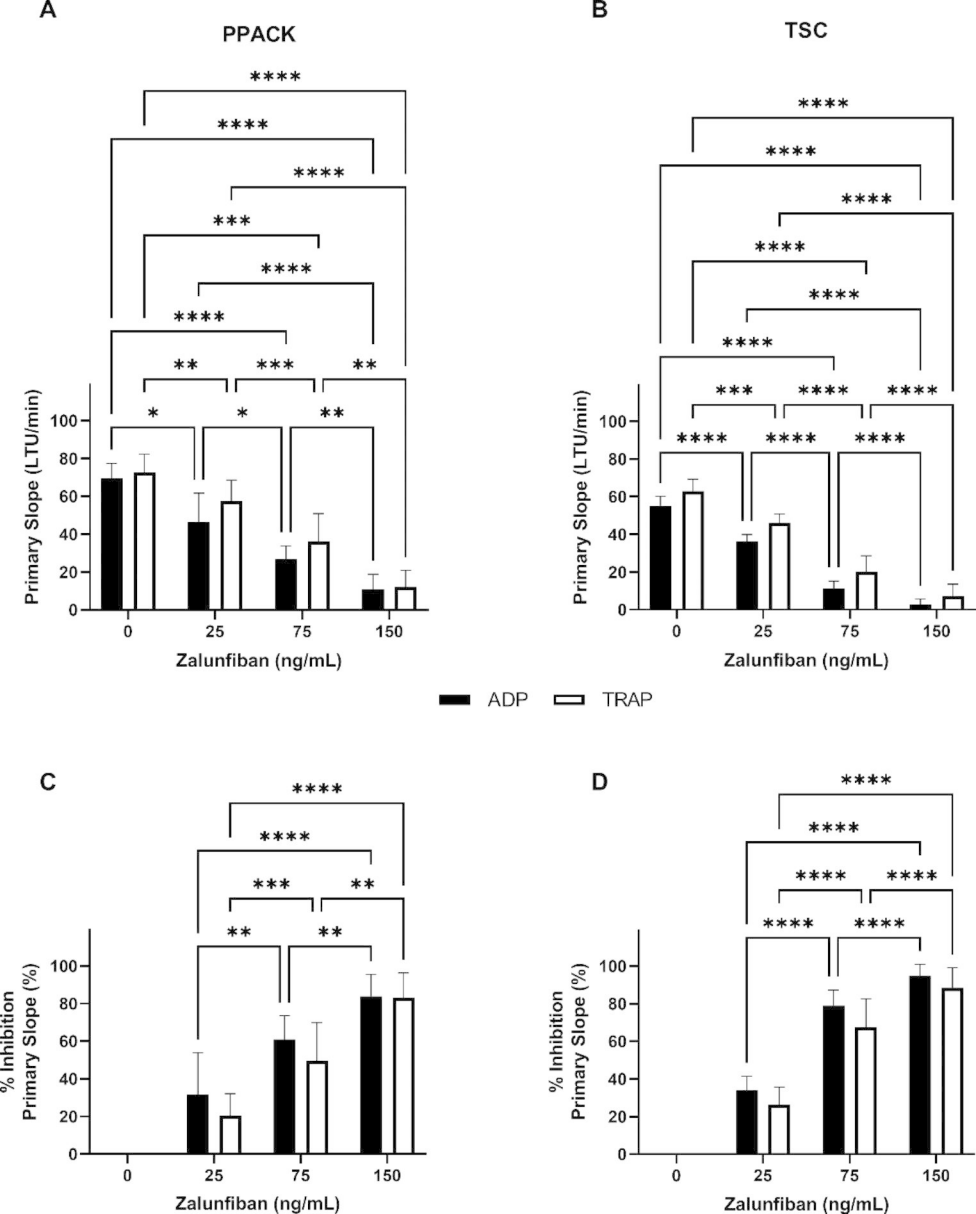
**Effects of zalunfiban treatment on rate of platelet aggregation**. PRP isolated from PPACK anticoagulated whole blood (A and C) or TSC anticoagulated whole blood (B and D) collected from healthy donors (n = 10) was treated with zalunfiban at the concentrations indicated. Platelet aggregation was initiated with ADP (black bars) or TRAP (white bars) agonists. Results are presented as the mean ± SD for primary slope (A and B) and percent inhibition of primary slope compared to vehicle control (C and D). Significance indicators are shown as *p ≤ 0.05, **p ≤ 0.01, ***p ≤ 0.001, and ****p ≤ 0.0001

After zalunfiban treatment, there was a progressive and marked decrease in PS with ADP and TRAP mediated aggregation in both PPACK and TSC anticoagulant (Fig. 2a and b). In PPACK anticoagulant at the reported Cmax zalunfiban concentration, the PS values for ADP and TRAP mediated aggregations were 10.9 ± 7.9 LTU/min (vehicle control 69.6 ± 7.8 LTU/min) and 12.0 ± 8.8 LTU/min (vehicle control 72.5 ± 9.8 LTU/min), respectively (Fig. 2a). The corresponding % inhibition of PS (compared to vehicle control) for ADP and TRAP mediated aggregations was 83.8 ± 11.7% and 83.1 ± 13.1%, respectively (Fig. 2c). The % inhibition of PS increased significantly with increasing concentrations of zalunfiban in response to both ADP and TRAP when tested in either PPACK and TSC anticoagulated PRP (Fig. 2c and d). The anticoagulant used for blood collection did not impact ADP or TRAP mediated aggregation responses at the zalunfiban concentrations tested, except at 75 ng/ml zalunfiban (*p < 0.01*; Table 1).

**Table 1:** Comparison of PPACK and TSC anticoagulants on platelet aggregation. PRP isolated from TSC- or PPACK-anticoagulated whole blood collected from healthy donors (n = 10) was treated with zalunfiban (25, 75 or 150 ng/mL) or selatogrel (50, 250 or 500 ng/mL) at the concentrations indicated. Platelet aggregation was initiated with ADP or TRAP agonists. Results are presented as the mean ± SD for percent inhibition of primary slope (top panel) and percent inhibition of maximal aggregation (bottom panel). Two-way ANOVA with Tukey’s multiple comparisons was performed between the two anticoagulants at each drug level. Significant differences are denoted in bold

Primary Slope (% Inhibition)		% Inhibition Primary Slope (Mean ± SD)			
Zalunfiban		TSC	PPACK	Adjusted P Value Primary Slope / % Inhibition	
ADP	25:TSC vs. 25:PPACK	34.1 ± 7.3	31.5 ± 22.3	*> 0.9977*	
	75:TSC vs. 75:PPACK	79.0 ± 8.2	60.9 ± 12.7	***0.0026***	
	150:TSC vs. 150:PPACK	94.9 ± 6.0	83.8 ± 11.7	*0.1485*	
TRAP	25:TSC vs. 25:PPACK	26.4 ± 9.2	20.4 ± 11.6	*0.8981*	
	75:TSC vs. 75:PPACK	67.5 ± 15.0	49.7 ± 20.2	***0.0148***	
	150:TSC vs. 150:PPACK	88.4 ± 10.7	83.1 ± 13.1	*0.9438*	
**Selatogrel**		**TSC**	**PPACK**	**Adjusted P Value** **Primary Slope / % Inhibition**	
ADP	50:TSC vs. 50:PPACK	10.6 ± 6.1	47.2 ± 19.8	***< 0.0001***	
	250:TSC vs. 250:PPACK	31.3 ± 10.1	65.8 ± 14.5	***< 0.0001***	
	500:TSC vs. 500:PPACK	37.1 ± 8.8	69.5 ± 13.9	***< 0.0001***	
TRAP	50:TSC vs. 50:PPACK	4.5 ± 4.0	10.1 ± 7.3	*0.6197*	
	250:TSC vs. 250:PPACK	10.4 ± 12.0	12.4 ± 7.6	*0.9977*	
	500:TSC vs. 500:PPACK	13.9 ± 19.2	13.8 ± 7.1	*> 0.9999*	
**Maximal Aggregation (% Inhibition)**		**% Inhibition Maximal Aggregation (Mean ± SD)**			
**Zalunfiban**		**TSC**	**PPACK**	**Adjusted P Value** **Primary Slope / % Inhibition**	
ADP	25:TSC vs. 25:PPACK	13.8 ± 9.1	12.6 ± 8.3	*> 0.9999*	
	75:TSC vs. 75:PPACK	64.7 ± 14.9	45.0 ± 22.6	***0.0007***	
	150:TSC vs. 150:PPACK	85.6 ± 9.4	76.4 ± 19.2	*0.3157*	
TRAP	25:TSC vs. 25:PPACK	12.4 ± 9.9	15.5 ± 18.6	*0.9996*	
	75:TSC vs. 75:PPACK	55.1 ± 25.5	40.5 ± 27.3	*0.3120*	
	150:TSC vs. 150:PPACK	82.6 ± 14.2	77.7 ± 21.2	*0.9926*	
**Selatogrel**		**TSC**	**PPACK**	**Adjusted P Value** **Primary Slope / % Inhibition**	
ADP	50:TSC vs. 50:PPACK	17.2 ± 14.9	60.1 ± 21.4	***< 0.0001***	
	250:TSC vs. 250:PPACK	51.3 ± 14.3	78.0 ± 11.2	***< 0.0001***	
	500:TSC vs. 500:PPACK	58.2 ± 10.8	80.5 ± 10.0	***< 0.0001***	
TRAP	50:TSC vs. 50:PPACK	10.8 ± 5.1	23.4 ± 15.4	***0.0024***	
	250:TSC vs. 250:PPACK	20.8 ± 11.9	27.2 ± 15.0	*0.3287*	
	500:TSC vs. 500:PPACK	20.3 ± 7.3	28.1 ± 13.2	*0.1354*	

**Fig. 3:**
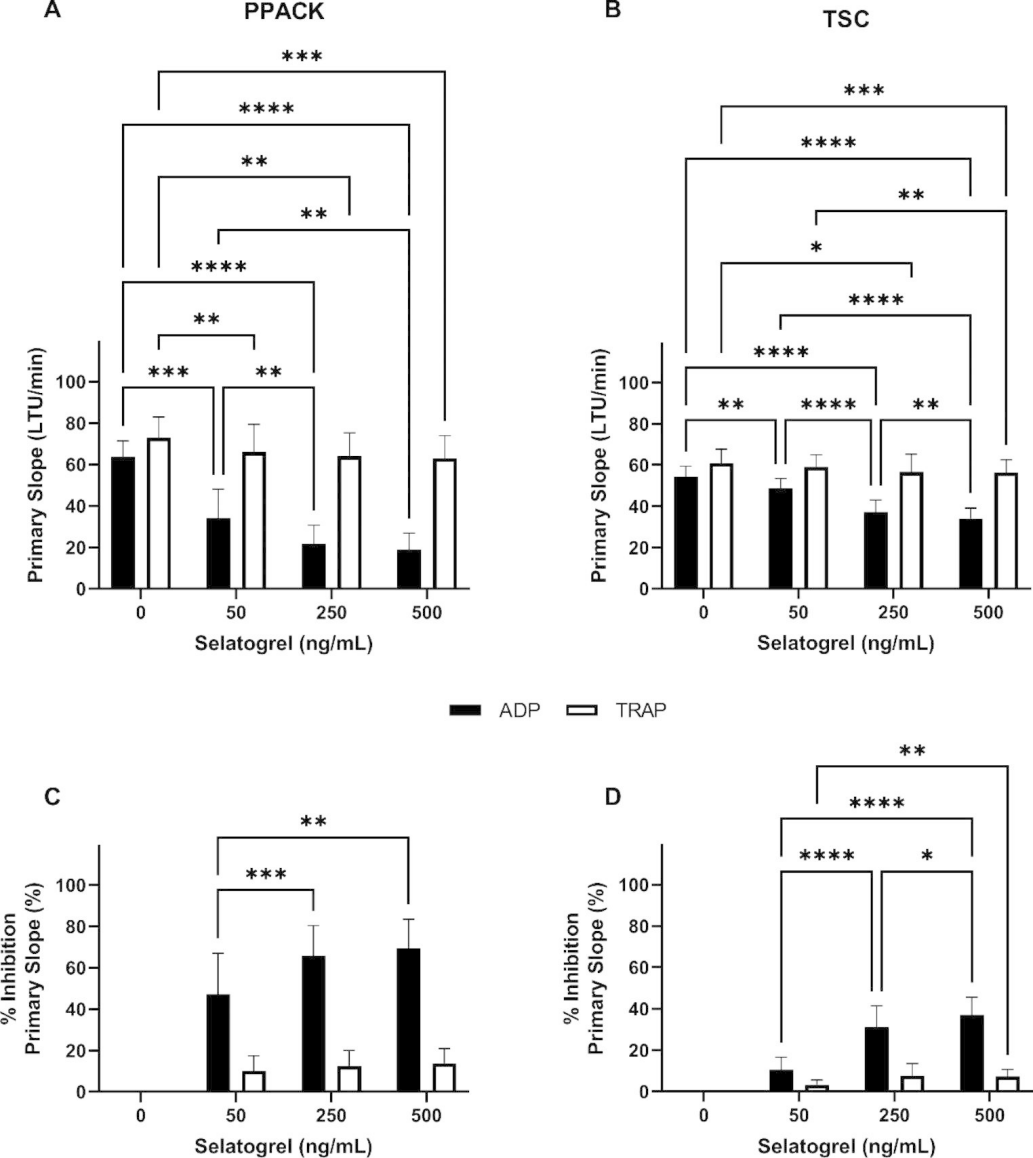
**Effects of selatogrel treatment on rate of platelet aggregation**. PRP isolated from PPACK anticoagulated whole blood (A and C) or TSC anticoagulated whole blood (B and D) collected from healthy donors (n = 10) was treated with selatogrel at the concentrations indicated. Platelet aggregation was initiated with ADP (black bars) or TRAP (white bars) agonists. Results are presented as the mean ± SD for primary slope (A and B) and percent inhibition of primary slope compared to vehicle control (C and D). Significance indicators are shown as *p ≤ 0.05, **p ≤ 0.01, ***p ≤ 0.001, and ****p ≤ 0.0001

After selatogrel treatment, PS response for ADP and TRAP mediated aggregations were confirmed to be significantly higher in TSC anticoagulated blood when compared to PPACK anticoagulated blood at all doses (Fig. 3; *p < 0.001*) and similar to earlier published data [23]. Due to this reported difference, data were primarily evaluated using blood collected in PPACK. At the reported Cmax selatogrel concentration, the PS values for ADP and TRAP mediated aggregations were 19.0 ± 7.8 LTU/min (vehicle control 63.8 ± 7.6 LTU/min) and 63.0 ± 11.0 LTU/min (vehicle control 72.9 ± 10.0 LTU/min), respectively (Fig. 3a). The corresponding % inhibition of PS (compared to vehicle control) for ADP and TRAP mediated aggregations was 69.5 ± 13.9% and 13.8 ± 7.1%, respectively (Fig. 3c). The % inhibition of PS increased significantly with increasing concentrations of selatogrel in ADP mediated aggregations between 50 and 250 ng/mL but not between 250 and 500 ng/ml selatogrel (Fig. 3c). The % inhibition of PS did not increase with increasing concentrations of selatogrel in TRAP mediated aggregations (*p > 0.05*; Fig. 3c).

**Fig. 4:**
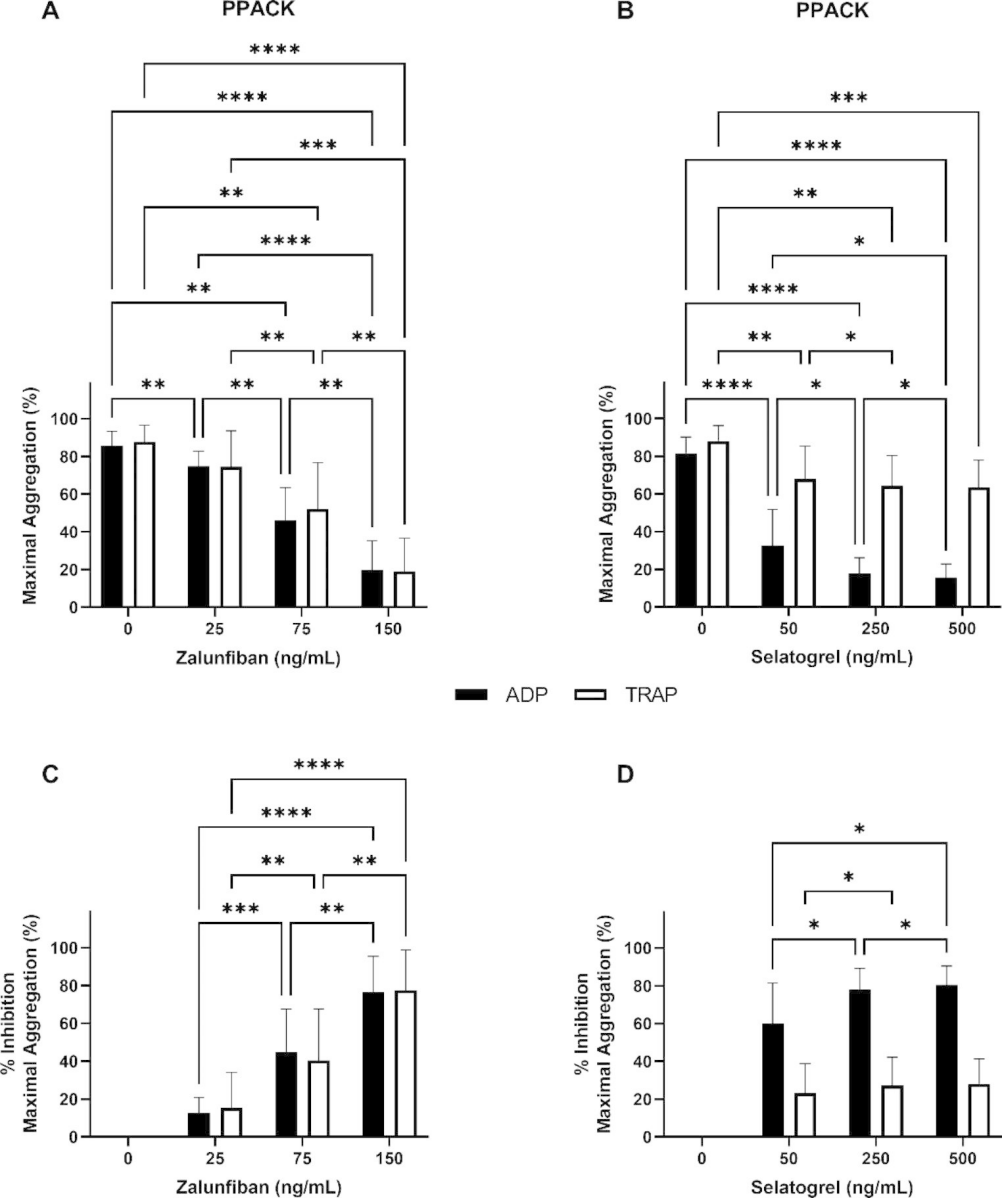
**Effects of zalunfiban and selatogrel treatment on the extent of platelet aggregation**. PRP isolated from PPACK anticoagulated whole blood collected from healthy donors (n = 10) was treated with zalunfiban (A and C) or selatogrel (B and D) at the concentrations indicated. Platelet aggregation was initiated with ADP (black bars) or TRAP (white bars) agonists. Results are presented as the mean ± SD for maximal aggregation (A and B) and percent inhibition of maximal aggregation compared to vehicle control (C and D). Significance indicators are shown as *p ≤ 0.05, **p ≤ 0.01, ***p ≤ 0.001, and ****p ≤ 0.0001

After zalunfiban treatment, there was a progressive and marked decrease in MA with ADP and TRAP mediated aggregations (Fig. 4a). At the reported Cmax zalunfiban concentration, the MA values for ADP and TRAP mediated aggregations were 19.8 ± 15.6% (vehicle control 85.8 ± 7.6%) and 19.1 ± 17.5% (vehicle control 87.8 ± 8.9%), respectively (Fig. 4a). The corresponding % inhibition of MA (compared to vehicle control) for ADP and TRAP mediated aggregations was 76.4 ± 19.2% and 77.7 ± 21.2%, respectively (*p < 0.0001*; Fig. 4c). The anticoagulant used for blood collection did not impact the % inhibition of MA in TRAP mediated aggregations, and the % inhibition of MA in ADP mediated aggregations was only significantly higher in TSC at 75 ng/mL zalunfiban (*p < 0.001*; Table 1).

After selatogrel treatment at the reported Cmax, MA response for ADP and TRAP mediated aggregations was 15.7 ± 7.2% (vehicle control 81.7 ± 8.5%) and 63.6 ± 14.3% (vehicle control 88.1 ± 8.2%), respectively (Fig. 4b). The corresponding % inhibition of MA for ADP and TRAP mediated aggregations was 80.5 ± 10.0% and 28.1 ± 13.2%, respectively (Fig. 4d). Similar to PS, there were significant differences between the % inhibition of MA in TSC vs. PPACK anticoagulant in ADP mediated aggregations at all selatogrel concentrations (*p < 0.0001*; Table 1) and only the 50 ng/mL concentration in TRAP mediated aggregations (*p < 0.003*; Table 1).

**Fig. 5:**
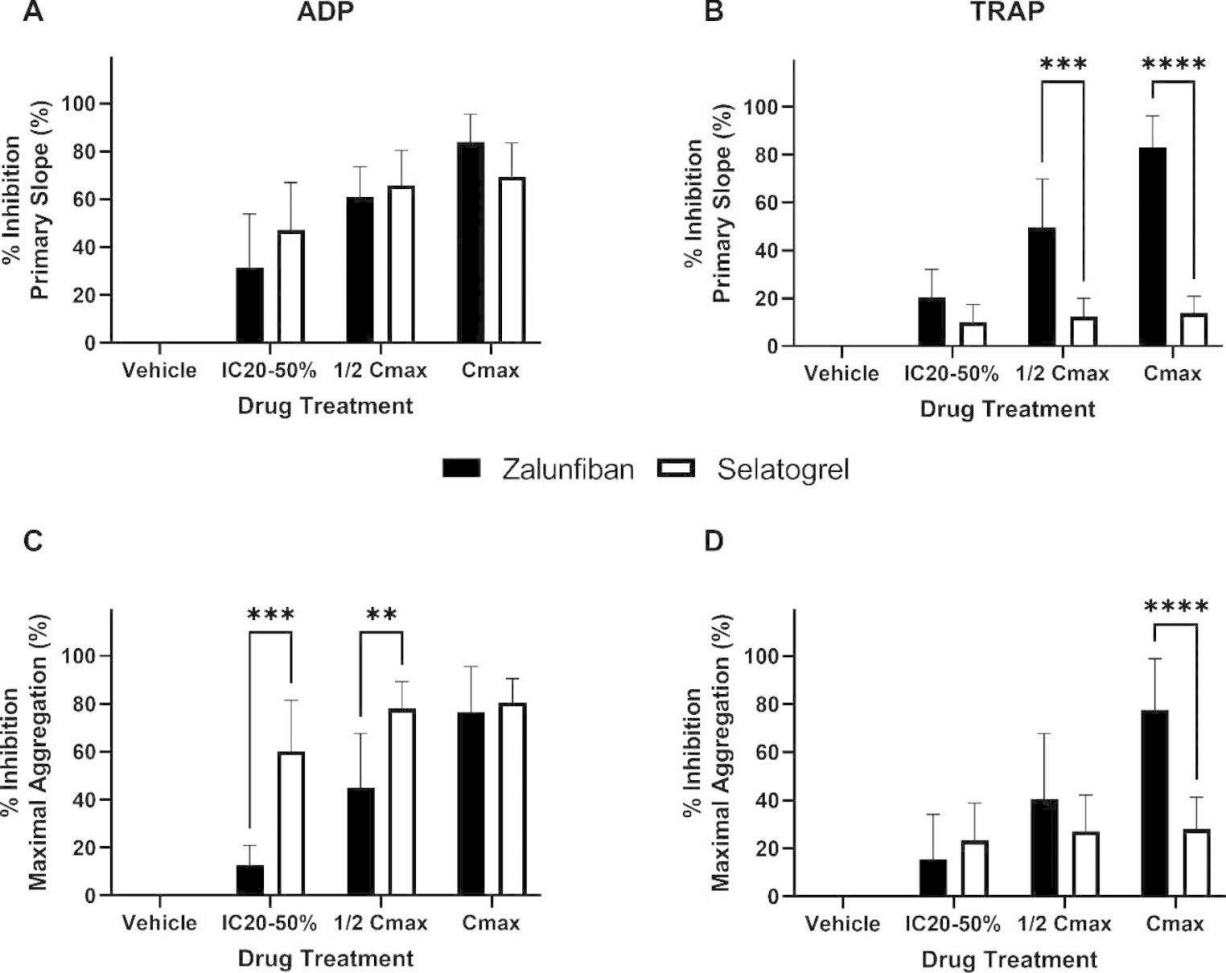
**Inhibitory effects of zalunfiban and selatogrel on the rate and extent of platelet aggregation**. PRP isolated from PPACK anticoagulated whole blood collected from healthy donors (n = 10) was treated with zalunfiban (black bars) or selatogrel (white bars) at the IC20-50%, ½ Cmax and Cmax concentrations from previous human clinical studies. Platelet aggregation was initiated with ADP (A and C) or TRAP (B and D) agonists. Results are presented as the mean ± SD for percent inhibition of primary slope compared to vehicle control (A and B) and percent inhibition of maximal aggregation compared to vehicle control (C and D). Two-way ANOVA with Šidák adjustment for multiple comparisons was performed between the two platelet inhibitors at each level. Significance indicators are shown as **p ≤ 0.01, ***p ≤ 0.001, and ****p ≤ 0.0001

When compared by IC20-50%, 1/2 Cmax, and Cmax, selatogrel and zalunfiban were equally effective in inhibition of PS in response to ADP mediated aggregations (Fig. 5a); however, zalunfiban demonstrated increased % inhibition of PS compared to selatogrel, specifically at the two higher doses (*p < 0.001* and *p < 0.0001*, respectively; Fig. 5b). Selatogrel % inhibition of PS in TRAP mediated aggregations averaged 12% across all three concentrations, whereas marked % inhibition of PS in TRAP mediated aggregations was observed with zalunfiban, especially at the 75 and 150 ng/mL concentrations (50% and 83%, respectively; Fig. 5b). Selatogrel was more effective in % inhibition of MA in ADP mediated aggregations at the IC20-50% and 1/2 Cmax concentrations compared to zalunfiban, but they were equivalent at the Cmax concentration (Fig. 5c). In contrast, zalunfiban was more effective in % inhibition of MA in TRAP mediated aggregations compared to selatogrel at the Cmax concentration (Fig. 5d). Selatogrel % inhibition of MA in TRAP mediated aggregations ranged from 23.4 to 28.1% across all doses compared to the zalunfiban % inhibition range of 15.5–77.7% (Fig. 5d).

## Discussion

A major finding of this study is that both zalunfiban and selatogrel were effective at inhibiting the extent of ADP mediated platelet aggregation but differed significantly in their capability to inhibit TRAP mediated platelet aggregation. While MA was lower in ADP mediated aggregations at the selatogrel low- and mid-level concentrations, the MA and corresponding % inhibition of MA were similar between the two drugs at concentrations corresponding to Cmax. Zalunfiban was comparable to selatogrel in decreasing PS in ADP mediated aggregations. In contrast, zalunfiban produced significantly lower PS and MA values in TRAP mediated platelet aggregations at all drug concentrations. These PD differences are due to the role that zalunfiban’s target receptor, GPIIb-IIIa, plays in mediating aggregation to all platelet agonists. That is, the capability of a GPIIb-IIIa antagonist to block fibrinogen binding upon platelet activation has the fundamental property of inhibiting platelet aggregate formation to all platelet primary (e.g., thrombin via PAR-1 and PAR-4 activation) and secondary agonists (e.g., ADP via P2Y12) compared to the single ADP pathway inhibition of a P2Y12 antagonist. The P2Y12 class of drugs that includes clopidogrel, prasugrel, cangrelor, ticagrelor and the investigational drug, selatogrel, specifically target one of the receptors that is activated by released ADP from the platelet dense granule. ADP is released from the granules upon activation by potent primary agonists such as collagen or thrombin and serves as a secondary agonist for GPIIb-IIIa-mediated platelet aggregation [29].

Two different anticoagulants were used for blood collection in our testing platforms. Previous reports indicated that both zalunfiban and selatogrel potencies differ when evaluated in TSC versus PPACK or heparin anticoagulated blood [27, 30]. These findings have been reported for other small molecule GPIIb-IIIa antagonists that had greater inhibition levels when blood was collected in TSC anticoagulants compared to non-calcium chelating anticoagulants such as PPACK or heparin [31, 32]. Therefore, PPACK anticoagulant was and is often recommended for PD evaluation of this drug class in clinical settings. Upon using a lower TSC concentration blood collection tube (3.2% vs. 3.8%), zalunfiban’s inhibition of MA in TRAP mediated aggregations mirrored that observed with PPACK anticoagulant for all drug concentrations tested. Differences between TSC and PPACK were noted at the 1/2 Cmax (75 ng/mL) when assessing % inhibition of PS in both ADP and TRAP mediated aggregations or when assessing % inhibition of MA in ADP mediated aggregations. Thus, the findings from our study suggest either PPACK or 3.2% TSC can be used when evaluating MA in TRAP mediated aggregations in the presence of zalunfiban. However, PPACK is recommended when evaluating MA in ADP mediated aggregations in the presence of zalunfiban or when evaluating PS in ADP or TRAP mediated aggregations.

In contrast, selatogrel demonstrated lower potency in our LTA testing when evaluating both PS and MA when TSC was used as the anticoagulant compared to PPACK and confirmed the earlier studies by Baldouni, et al. [27]. Our data demonstrated that selatogrel PD assessments require testing in PPACK anticoagulant to measure its potency to inhibit ADP mediated aggregation. It is not recommended that selatogrel % inhibition of PS and MA in TRAP mediated aggregations be measured, as both criteria were relatively low and similar for all concentrations tested (< 15% and < 30%, respectively).

Two platelet function parameters were evaluated in this study: the rate of aggregate formation (PS) and extent of aggregate formation (MA). PS may be the most clinically relevant measure for thrombin mediated events as its generation occurs rapidly in STEMI patients, and the resultant fibrin formation has a direct correlation to aggregate formation and stability [33]. A significant association has been reported between procoagulant activity, especially by D-dimer and prothrombin fragment 1 + 2, markers of thrombin generation and activity, and impaired myocardial function in the acute phase of STEMI [34]. Consequently, a key difference between the two inhibitors was the PS measured, using the thrombin mimic, TRAP, to mediate the aggregation response where zalunfiban had greater % inhibition of PS compared to selatogrel.

Upon review of the PD properties, selatogrel, in contrast to the oral P2Y12 inhibitors, demonstrated a faster onset of ADP-mediated platelet aggregation inhibition within 15 min of administration and maintained significant inhibition up to 8 h after administration, returning to near pre-dose levels by 24 h [25]. In comparison, the initial dose evaluation of zalunfiban had a similar onset of platelet inhibition within 15 min of administration, with 50% of platelet function returning between 90 and 120 min post injection [24]. As thrombin serves as a primary agonist and leads to platelet activation, including the secondary release of ADP from internal storage granules, early harnessing of the rate of thrombus formation and inhibiting fibrinogen binding with nearly complete GPIIb-IIIa receptor blockade may be more efficacious for improving outcomes of patients with STEMI. In patients at increased risk for the occurrence of ischemic events, inhibition of P2Y12 even by the more potent inhibitors, may not be sufficient, as activation via PAR-1 and PAR-4 is preserved. Thrombin, the most potent platelet agonist, is generated within seconds of a vascular injury such as plaque rupture and primarily generated on the surface of the activated platelet. Thus, the data support that zalunfiban provides additional benefit in blocking the activity of thrombin and secondary agonists and in reducing the rate and extent of aggregation formation.

Anti-platelet agents such as aspirin and an oral P2Y12 antagonist, have been shown to improve patient outcomes with ACS, including STEMI [35]. However, the parenteral GPIIb-IIIa inhibitors offer more rapid and broader platelet inhibition against both primary (thrombin) and secondary agonists (ADP), as demonstrated by platelet function testing [31]. Previous studies demonstrated that the administration of parenteral GPIIb-IIIa antagonists was associated with higher patency of the culprit artery, resulting in ST-elevation resolution and improvement in the rate of perfusion [36–38]. Multiple studies have shown that pre-procedural primary PCI therapy with a GPIIb-IIIa antagonist can increase pre-procedure infarct artery blood flow, speed ST-segment resolution, contain infarct size, and improve STEMI patient survival [39–42]. Despite these benefits, upstream use of parenteral GPIIb-IIIa is not considered standard of care for STEMI, largely due to the need for parenteral administration and the bleeding risks associated with first-generation GPIIb-IIIa inhibitors [43]. However, the availability of a shorter-duration, injectable pre-hospital GPIIb-IIIa inhibitor may satisfy the need for imperative coronary reperfusion in a timely manner in this patient population.

In conclusion, zalunfiban and selatogrel represent a new generation of platelet antagonists that may be administered subcutaneously at first medical contact in patients with STEMI. Although in vitro spiking studies may not directly translate to the clinical setting, and thus additional ex vivo pharmacokinetic and PD studies may be needed, the present findings warrant further investigation into the potential benefits of the short-acting GPIIb-IIIa inhibitor zalunfiban for patients at risk for further ischemic events. The ongoing phase III double-blinded, randomized, placebo-controlled trial (CeleBrate), will provide more definitive answers and shed further light on the role of these inhibitors in improving outcomes for such patients (NCT04825743) [44].
